# Atractylodes Japonica Rhizome Extract Fermented with a Plant-Derived *Lacticaseibacillus paracasei* (*Lactobacillus paracasei*) IJH-SONE68 Improves the Wheat Gliadin-Induced Food Allergic Reaction in Mice

**DOI:** 10.3390/nu17071151

**Published:** 2025-03-26

**Authors:** Qingmiao Ma, Masafumi Noda, Narandalai Danshiitsoodol, Masanori Sugiyama

**Affiliations:** Department of Probiotic Science for Preventive Medicine, Graduate School of Biomedical and Health Sciences, Hiroshima University, Kasumi 1-2-3, Minami-ku, Hiroshima 734-8551, Japan; ma1202qingmiao@gmail.com (Q.M.); bel@hiroshima-u.ac.jp (M.N.); naraa@hiroshima-u.ac.jp (N.D.)

**Keywords:** lactic acid bacteria, *Lacticaseibacillus paracasei*, Atractylodes Japonica Rhizome, wheat gliadin, food allergic reaction

## Abstract

**Background/Objectives:** Medicinal herbs produce valuable substances with therapeutic potential. The chemical structures of those substances are often converted by gut microbiota. Our previous studies showed that several kinds of bioactive molecules are newly generated in fermented medicinal herbal extract with plant-derived lactic acid bacteria (LABs). **Methods:** The fermented extract of Atractylodes Japonica Rhizoma (AJR), which is designated as “Byakujutsu” in Japan, with a plant-derived LAB strain IJH-SONE68 was prepared and whether the fermented extract could help reduce symptoms of food allergies, especially wheat intolerance, was confirmed using animal model. **Results:** It has been found that the fermented extract significantly ameliorates the anaphylaxis score (from 3.0 to 1.0, *p* = 0.003) of gliadin-induced allergic model mice (specific-pathogen-free, BALB/cJ) accompanied with the modulation of serum total immunoglobulin E (IgE) (from 778 to 518 ng/mL, *p* = 0.006), interferon (IFN)-γ (from 6.6 to 9.5 pg/mL, *p* < 0.001), and interleukin (IL)-4 (from 32.0 to 9.1 pg/mL, *p* < 0.001) levels. **Conclusions:** The fermented AJR extract may modulate the Th1/Th2 cell balance to alleviate the symptoms of gliadin-induced anaphylaxis in mice. The present study supports the view that the fermentation of medicinal herbal extract prepared using LABs may be a useful procedure for producing therapeutic potential compounds to maintain health.

## 1. Introduction

Wheat is recognized as one of the important grains consumed in the world, whereas the wheat can also cause severe allergic reactions. The number of people with wheat allergies has increased over the past decade [1]. Both adults and children can experience different symptoms of wheat allergy. Children are more likely to develop atopic eczema/dermatitis syndrome (AEDS), while adults more commonly experience wheat-dependent exercise-induced anaphylaxis (WDEIA) [2,3,4]. Gliadins, which are wheat-derived major prolamin proteins that have poor solubility in water, were reported first as an allergen causing WDEIA. The compound ω-5 gliadin is known to be a major allergen responsible for WDEIA in adults and immediate wheat-induced anaphylaxis in young children [5,6,7,8,9,10].

Atractylodes Japonica Rhizoma (AJR), which is known as “Baizhu” in China and “Byakujutsu” in Japan, is the rhizome of *Atractylodes japonica* Koidzumi [11]. The broad pharmacological effects of AJR on various disease have been reported to improve gastrointestinal function, as well as immunomodulatory, anti-inflammatory, anti-bacterial, and anti-tumor activities [12]. Although several anti-inflammatory constituents of AJR have been determined, and some of those have been reported to inhibit systemic and passive cutaneous anaphylaxis reactions in model mice [13,14,15], the beneficial effects of AJR on food allergy remain unclear and require further study.

Medicinal herbs contain valuable therapeutic potential molecules [16], acting as natural resources of bioactive compounds like dietary fibers, antioxidants, phenols, glycosides, minerals, and vitamins. In general, gut microbiota can biotransform these plant-derived substances into bioactive ones that display their therapeutic effects [17], this means that the intestinal bacteria play a crucial function in maximizing the benefits of these herbs by the bioconversion of complex substances into more compatible components through fermentation. This process can improve the bioavailability and biological activity of phytochemicals and lead to a significant increase in functional microbial metabolites, resulting in beneficial effects on human health [18]. In fact, some bioactive molecules have been found in fermented medicinal herbal extracts through our previous studies by fermentation technique using plant-derived lactic acid bacteria (LABs) [19,20,21,22].

Based on these findings, we have focused on whether the fermented AJR extract with LABs could help reduce symptoms of food allergies, especially wheat intolerance. In this study, we used a mouse model of sensitive to gliadin to evaluate the therapeutic effects of the combination of AJR and our LAB isolate, *Lacticaseibacillus paracasei* (formerly *Lactobacillus paracasei*) IJH-SONE68, which was first found as exopolysaccharide (EPS)-producing strain [23] and can grow in the AJR extract vigorously.

## 2. Materials and Methods

### 2.1. Preparation of AJR Extract and LAB Culture Condition

The rhizome of *A. japonica* was obtained from Kojima Kampo (Osaka, Japan) in a dried, cut form. The rhizome pieces were mixed with distilled water to a final concentration of 7.5% (*w*/*v*) and heat-treated at 105 °C for 30 min. The solid residues were removed by centrifugation, and the resulting supernatant was adjusted to pH 7.0 using NaOH solution. After sterilizing the solution at 120 °C for 15 min, the AJR extract was used as a cultivation medium for LABs. The AJR extract without LAB fermentation was also directly used as an unfermented AJR extract in animal experiments.

For seed cultivation, *L. paracasei* IJH-SONE68 was grown as standing culture in de Man, Rogosa, and Sharpe (MRS) broth (Merck KGaA, Darmstadt, Germany) at 28 °C. The seed culture was then added to the above AJR extract at a ratio of 1% (*v*/*v*), followed by incubation at the same temperature for 24 h under the same condition. After fermentation, the cultured broth was sterilized at 120 °C for 20 min, and bacterial cell debris was removed by centrifugation. The resulting supernatant was used as the fermented AJR extract.

### 2.2. Extraction and Preparation of Wheat Gliadins

The gliadin fraction was extracted according to the procedure of previous report [24]. Briefly, 50 g of wheat flour “Flower” (Nisshin Flour Milling Inc., Tokyo, Japan) was mixed with 10 volumes of 0.5 M sodium chloride solution at room temperature for 2 h to extract salt-soluble proteins. After centrifugation (1700× *g*, 15 min), five-hundred ml of distilled water was added to the residue, and the water-soluble albumins and salts were extracted into the water by stirring at room temperature for 2 h, and the residue was collected by centrifugation in the same conditions. After repeating the same extraction process twice, 500 mL of 70% (*v*/*v*) ethanol was added to the residue, and the gliadins were extracted by stirring at 4 °C for 2 h. The ethanol fraction obtained by centrifugation under the same condition was poured into the dialysis membrane and dialyzed against 1% (*v*/*v*) acetic acid solution at 4 °C for 60 h. During the dialysis, the acetic acid solution was changed every two to three hours on the first day, and then two or three times a day after the second day. The lyophilized dialysate was used as a gliadin fraction.

### 2.3. Animal Treatment and Sensitization by Gliadin

Seven-week-old female specific-pathogen-free (SPF) BALB/cJ mice were obtained from Shimizu Laboratory Supplies, Co., Ltd. (Kyoto, Japan). They were housed in plastic cages under controlled conditions: a temperature of 20–26 °C, humidity of 40–60%, and a 12-h light/dark cycle. The mice were fed an MF rodent diet (Oriental Yeast Co., Ltd., Tokyo, Japan) ad libitum and had unlimited access to water. The animal experiments were performed at Institute of Laboratory Animal Sciences of Hiroshima University following the university’s “Guidelines for the Care and Use of Laboratory Animals”. The mice were marked with different colored felt markers (Animal Marker, Muromachi Kikai Co., Ltd., Tokyo, Japan) on their tails. The study protocol was approved by the Laboratory Animal Science Research Facility Committee of Hiroshima University (Permit Number: A22-156) before conducting the experiment.

After a week of familiarization, the mice were randomly divided into four experimental groups of five as follows: the without-sensitization group (negative control, NC), the sensitized-but-not-treated group (sensitization-positive control, PC), the sensitized and unfermented AJR-treated group (Unf-AJR), and the sensitized and fermented AJR-treated group (Fer-AJR). The race of mice and sample size were determined based on our previous studies that revealed the anti-inflammatory properties of the IJH-SONE68 strain [25,26]. Each mouse was given 100 μL portion of 10 mg/mL gliadin on days 3, 7, and 11 by oral feeding for immunization. According to the sensitization method of Shindo et al. [27], gliadin was dissolved in the mixture of linoleic acid/lecithin (4:1) containing 0.75% (*v*/*v*) ethanol and 3 mg/mL sodium salicylate.

Throughout the experiment, the AJR extract (fermented or unfermented) was given daily as a 100 μL oral dose, while the NC and PC groups received the sane volume of sterile water. On day 14, the mice were challenged with a 100-fold dose of gliadin. On the last day of the experiment (day 15), the mice were euthanized using isoflurane anesthesia, and blood samples were collected. Serum levels of total immunoglobulin E (IgE), interferon (IFN)-γ, and interleukin (IL)-4 were measured using enzyme-linked immunosorbent assay (ELISA) Kits (BioLegend, San Diego, CA, USA) in accordance with the manufacturer’s instructions.

In the present experimental plan, the study was conducted with the consideration that, from a humanitarian perspective, euthanasia could be an option for individuals exhibiting severe tremors or significant debilitation due to intoxication or allergic reactions. The order of treatments and measurements for each group was not predetermined and was conducted randomly each time. In addition, cage location was unintentionally assigned to available address upon the arrival of animals at the experimental facility, thus there was no arbitrariness.

### 2.4. Quantitative Evaluation of Symptoms in Gliadin-Challenged Mice

After a 1-h period of challenging, the anaphylaxis symptoms observed in each mouse were assessed using a scoring system as follows [28]: asymptomatic individuals were given a score of 0; scratching the mouth and ears with a hind leg or ruffling the fur was scored as 1; nausea was scored as 2; labored respiration or cyanosis of the mouth and tail was scored as 3; and serious symptoms leading to death within 24 were scored as 4.

### 2.5. Purification of EPS from Culture Supernatant

EPS purification from culture supernatant was carried out as described previously [29]. Briefly, after cultivation of IJH-SONE68 strain using a modified semi-defined medium [29], the cultured broth was treated with trichloroacetic acid (TCA) and centrifuged to remove the LAB cell mass and proteins. The obtained supernatant was mixed with an equal volume of acetone, and then the precipitated crude EPS was collected by centrifugation. To further purify the EPS, the crude product was dissolved into appropriate buffer and treated with deoxyribonuclease I (Worthington Biochemical Corporation, Lakewood, NJ, USA), ribonuclease A (Nacalai Tesque, Kyoto, Japan), and proteinase K (Wako Pure Chemical Industries, Ltd., Osaka, Japan) to eliminate any remaining nucleic acids and proteins. The protein debris were removed through TCA precipitation, and the EPS was collected by ethanol precipitation from the resultant supernatant. The obtained EPS was dissolved in distilled water and ultrafiltrated using an Amicon Ultra centrifugation unit (MWCO = 10 kDa, Merck Millipore Ltd., Carrigtwohill, Co., Cork, Ireland) to remove salts and small size polymers. The EPS content was determined by the phenol sulfate method [30].

### 2.6. Statistical Analyses

GraphPad Prism 8 software (GraphPad Software, Inc., Boston, MA, USA) was used for conducting all statistical analyses. For multiple comparison analyses, the Tukey–Kramer test was conducted [31]. If not applicable, the Steel–Dwass test was used instead [32]. The person who performed the procedures on the animals was different from the person who conducted group allocation and statistical analyses.

## 3. Results

### 3.1. Ameliorative Effect on Gliadin-Induced Allergic Symptoms

**Figure 1:**
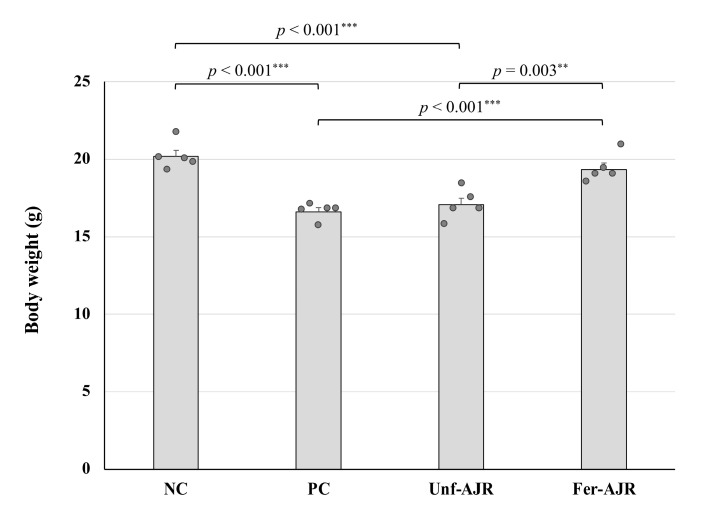
A comparison of body weight in gliadin-induced anaphylaxis model mice. The mice were divided into four experimental groups of 5 mice each as follows: the without-sensitization group (the negative control), NC; the sensitized-but-not-treated group (the sensitization positive control), PC; the sensitized and unfermented AJR-treated group, Unf-AJR; and the sensitized and fermented AJR-treated group, Fer-AJR. Statistical analyses were conducted using the Tukey–Kramer method (** *p* < 0.01, *** *p* < 0.001). The values are presented as the mean ± standard error (S.E.).

During the experimental period, there were no animals excluded from the analyses. Body weight losses were observed in all mice sensitized with gliadin, especially in the group without treatment (the sensitization-positive control, PC) with statistical significance (*p* < 0.001) against the group without sensitization (the negative control, NC) (Figure 1). The amelioration of body wight loss was not observed in mice treated with unfermented AJR extract (Unf-AJR) but present in the fermented AJR-treated group (Fer-AJR) with statistical significance (*p* = 0.003).

**Figure 2:**
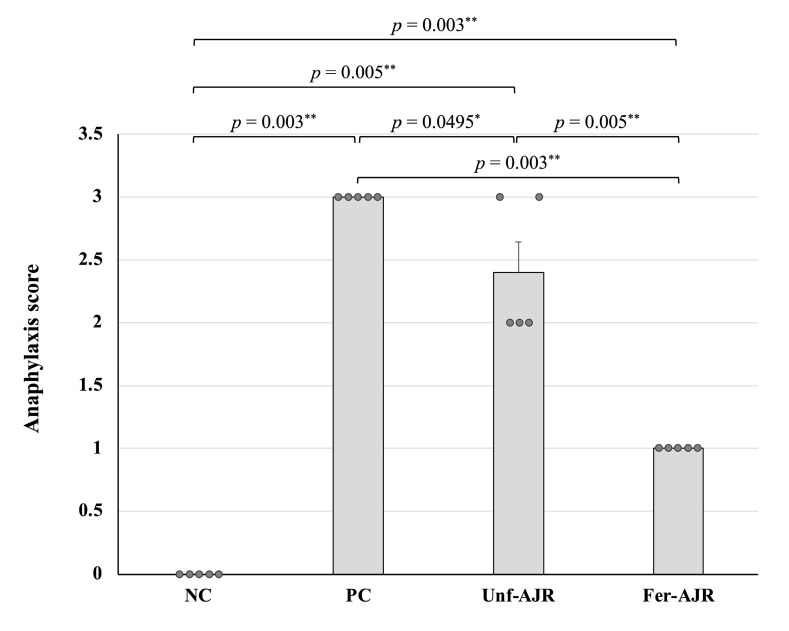
The observed anaphylaxis score within 24 h of the gliadin challenge. Abbreviations are the same as in Figure 1. Statistical analyses were conducted using the Steel–Dwass test (* *p* < 0.05, ** *p* < 0.01). The values are presented as the mean ± S.E.

After gliadin challenge, no measurable changes were observed in the NC group, whereas remarkable anaphylactic reactions occurred in the PC and Unf-AJR groups (Figure 2). In contrast, the feeding of the fermented AJR significantly (*p* = 0.003) ameliorated the anaphylaxis symptoms as compared with the PC group; specifically, mice displayed only slight symptoms in the Fer-AJR group.

**Figure 3:**
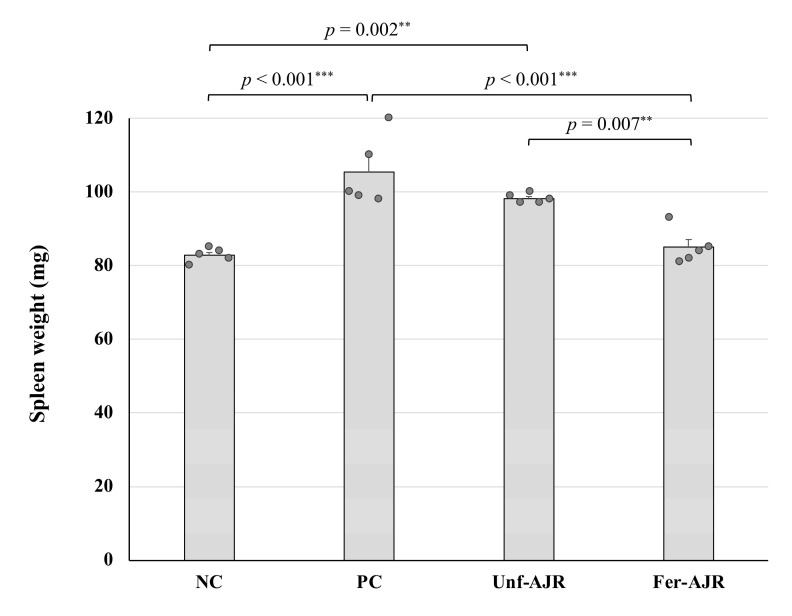
A comparison of spleen weight in model mice. Abbreviations are the same as in Figure 1. The values are presented as the mean ± S.E. Statistical analyses were conducted using the Tukey–Kramer method (** *p* < 0.01, *** *p* < 0.001).

Additionally, an analysis of the average spleen weight in each group showed that the severity of the allergic symptoms conversely correlates with the spleen enlargement (Figure 3), which has been reported to be observed in mice with dextran sulfate sodium (DSS)-induced colitis and ovalbumin (OVA)-induced food allergy [33,34,35].

### 3.2. Repressive Effect on the Serum Total IgE Level

**Figure 4:**
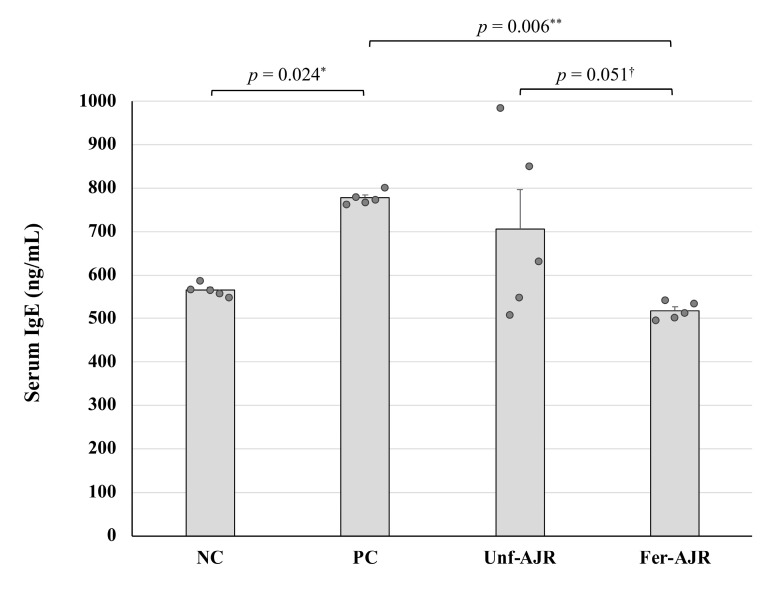
The differences in the serum IgE levels of the model mice. Abbreviations are the same as in Figure 1. Statistical analyses were conducted using the Tukey–Kramer method (^†^ *p* < 0.1, * *p* < 0.05, ** *p* < 0.01). The values are presented as the mean ± S.E.

Because allergic responses observed in gliadin-induced anaphylaxis are IgE-mediated and accompanied by the IgE-dependent degradation of mast cells [36], the serum total IgE levels of each mouse were measured and compared across all groups (Figure 4). The mean IgE concentration in the PC group was significantly higher than that in NC and Fer-AJR groups (*p* = 0.024 and *p* = 0.006, respectively), reflecting allergic symptoms, whereas unfermented AJR seemed to be ineffective at improving the IgE level.

### 3.3. Fermented AJR Regulates Th1 and Th2 Cell Responses

**Figure 5:**
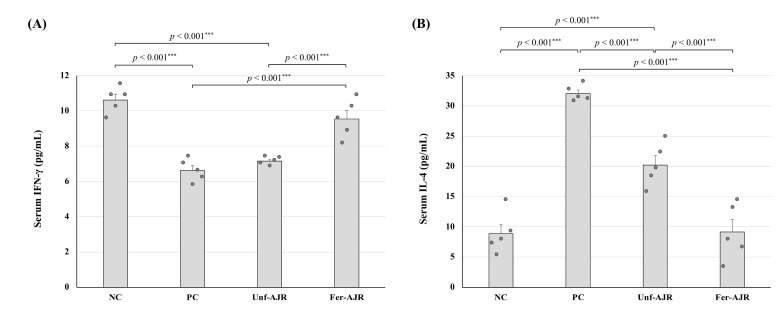
Differences in the concentrations of serum cytokines IFN-γ (**A**) and IL-4 (**B**) of the model mice. Abbreviations are the same as in Figure 1. Statistical analyses were conducted using the Tukey–Kramer method (*** *p* < 0.001). The values are presented as the mean ± S.E.

Generally, food allergies are triggered by an imbalance of the Th1/Th2 response ratio; thus, inordinate Th2-type immune responses can be used to evaluate allergic symptoms [37,38]. To confirm whether the Th1/Th2 balance was altered, serum concentrations of cytokines IFN-γ and IL-4, which were Th1- and Th2-type cytokines, respectively, were determined (Figure 5). The PC group of mice had significant decreases and increases in IFN-γ (*p* < 0.001) and IL-4 (*p* < 0.001) levels, respectively, as compared with those of the NC group. While these altered cytokine levels were almost restored by treatment with the fermented AJR extract, only a partial improvement in the IL-4 level was observed in the Unf-AJR group.

## 4. Discussion

There are no significant progresses in grasping the role of LABs and other beneficial microbes in a wheat-induced imbalance in the mucosal ecosystem. In general, food-derived protein-induced allergic reactions are initiated in the intestinal mucosa, where the immune response is accompanied by mucosal disease, intestinal tissue damage, and cytokine production [39]. In addition to host gastrointestinal cells, imbalances in the gut microbiota of patients with allergic diseases have been widely reported, such as decreases in Lactobacillaceae, *Synechococcus*, and *Clostridium*, and changes in intestinal microbiota diversity [40,41]. Overall, probiotics have been noted as potential alternative therapies. Current reports suggest that probiotics may contribute to intestinal barrier function repair, intestinal flora restoration, antigen modification, and systemic immune modulation [41,42,43,44,45]. However, the exact anti-allergic mechanisms of probiotic therapy have not been fully understood, which limits their broader clinical applications. Therefore, further research is needed to identify specific beneficial microbes like probiotics for different types of allergies [46].

For understanding the mechanisms of sensitization and allergic symptom induction, relevant animal models may help us. Because the murine immunology has been well understood and well characterized, a mouse model has been widely used to study the immune responses to Th1, Th2, or Th17 phenotypes. Schematically, IL-4 and IL-13 secreted by Th2 cells induce IgE production in mice, whereas IFN-γ secreted by Th1 cells induces T cell–mediated immunity and down-regulates Th2 cells [47]. Th17 cells have been described as being associated with autoimmune diseases [48]. BALB/c mice, which are a Th2-biased strain with high IgE response, have been widely used for sensitization to food allergens to obtain specific IgE responses after intragastric and intraperitoneal administration [49,50,51,52]. The administration of bovine β-lactoglobulin or ovalbumin into BALB/c mice induces IgE responses that are specific to the same epitopes involved in allergy patients [40,49]. This strain has also been used to study the early and late stages of anaphylaxis after intraperitoneal sensitization. Relationships between the structural characteristics of the protein and the pathophysiology of allergic reactions have been clarified [51].

Although the spleen is the largest lymphoid organ, it plays multiple roles in interacting with various blood cells [53,54,55]. The systemic blood in the body directly interacts with the spleen, where it is continuously monitored by immune cells. As a result, both innate and adaptive immune responses are initiated in the spleen [56,57,58]. During an immune response, the activation of T and B cells cause the proliferation of splenic cells, resulting in the spleen enlargement [59]. The spleen enlargement was also often observed in not only mice suffered from inflammatory disorders but also food allergy model mice [33,34,35,60,61,62,63], indicating that an excess immune response may lead to abnormal spleen size. In the present study, gliadin-sensitized mice showed the increase in spleen size, however, the size was reduced in accordance with anaphylaxis score by administration of fermented-AJR extract. Therefore, the LAB-fermented AJR extract may modulate immune responses.

AJR, a herbal medicine traditionally used for digestive disorders and rheumatism, is composed of a few essential ingredients, including sesquiterpenes, phenolic acids, and polyethylene alkynes [64]. For instance, atractylodin, which is a polyethylene alkyne component of *A. japonica*, has been found to improve intestinal inflammation by regulating mitogen-activated protein kinase (MAPK) and reduce acute lung injury by inhibiting nucleotide binding domain-like receptor protein 3 inflammasome and Toll-like receptor 4 activation [63,64,65,66,67,68]. Recent studies have shown that atractylodin ameliorates collagen-induced arthritis in mice by modulating the maturation of dendritic cells [69]. Additionally, it has been reported that atractylodin suppresses the Th2 response by regulating dendritic cells, leading to a decrease in the concentration of IL-4. As a result, the Th1 response is promoted followed by increasing the concentration of IFN-γ [70]. On the other hand, it has been reported that the oral administration of IJH-SONE68 strain–derived EPS [23] inhibits the acceleration of IL-4 and serum IgE expression in model mice with delayed-type allergies [25].

**Figure 6:**
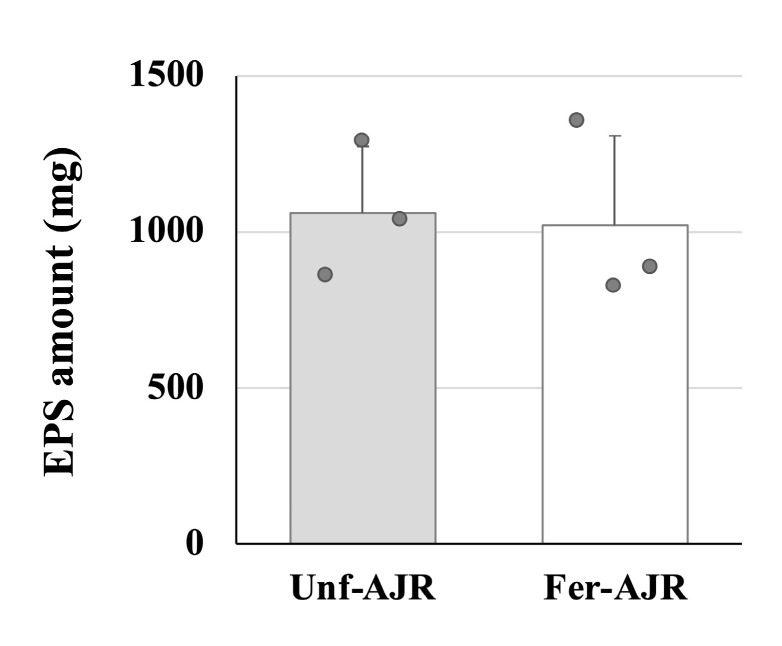
The comparison of the sugar concentration of the EPS fraction purified from 5 mL of unfermented (Unf-) and fermented (Fer-) AJR extract with IJH-SONE68 strain.

The fermented AJR extract contains soluble components of IJH-SONE68 strain cells including those released after sterilization, thus there are possibilities that those components might influence the outcomes of the animal experiment. However, previous studies have confirmed that the observed anti-inflammatory effects and immunomodulatory activities of IJH-SONE68 strain are primarily attributed to the EPS [25,26]. Therefore, to confirm whether the EPS is also produced in the fermented AJR extract, we have measured and compared the polysaccharides’ contents purified from the AJR extract before and after fermentation with the IJH-SONE68 strain (1.1 ± 0.2 and 1.0 ± 0.3 mg yields from 5 mL samples, respectively) (Figure 6). The result shows that the production of EPS was not satisfactory during the fermentation of the AJR extract, indicating that the ameliorative effects on gliadin-induced allergic model mice observed in the present study may be due to specific metabolites produced during fermentation rather than EPSs. In fact, unlike the fermented AJR extract, the EPS derived from the IJH-SONE68 strain exhibited its anti-inflammatory effect not through Th1 upregulation but through Th2 downregulation [25].

## 5. Conclusions

How the fermented AJR extract modulates the Th1/Th2 cell balance and/or alleviates the symptoms of food allergy in mice is still unclear due to the insufficiency of molecular biological data. While further analyses are currently underway to identify the specific substances contributing to the observed ameliorative effects, our results suggest that the fermentation of medicinal herbs with LABs holds significant potential for producing active compounds with therapeutic potential and promoting preventive healthcare.

## Data Availability

The original contributions presented in the study are included in the article; further inquiries can be directed to be corresponding author.

## Abbreviations

The following abbreviations are used in this manuscript:

AEDS	atopic eczema/dermatitis syndrome
AJR	Atractylodes Japonica Rhizoma
DSS	dextran sulfate sodium
ELISA	enzyme-linked immunosorbent assay
EPS	exopolysaccharide
IFN	interferon
IgE	immunoglobulin E
IL	interleukin
LAB	lactic acid bacterium
MAPK	mitogen-activated protein kinase
MRS	de Man, Rogosa, and Sharpe
OVA	ovalbumin
SPF	specific-pathogen-free
TCA	trichloroacetic acid
WDEIA	wheat-dependent exertion-induced anaphylaxis
